# Cell-Penetrating Milk-Derived Peptides with a Non-Inflammatory Profile

**DOI:** 10.3390/molecules28196999

**Published:** 2023-10-09

**Authors:** Clement Agoni, Ilias Stavropoulos, Anna Kirwan, Margharitha M. Mysior, Therese Holton, Tilen Kranjc, Jeremy C. Simpson, Helen M. Roche, Denis C. Shields

**Affiliations:** 1 Conway Institute of Biomolecular and Biomedical Research, University College Dublin, Belfield, D04 V1W8 Dublin 4, Irelandmargaritha.mysior@ucd.ie (M.M.M.); jeremy.simpson@ucd.ie (J.C.S.);; 2 School of Medicine, University College Dublin, Belfield, D04 W6F6 Dublin 4, Ireland; 3 Discipline of Pharmaceutical Sciences, University of KwaZulu Natal, Durban 4041, South Africa; 4 School of Biology and Environmental Science, University College Dublin, Belfield, D04 N2E5 Dublin 4, Ireland; 5 Institute of Food and Health, University College Dublin, Belfield, D04 V1W8 Dublin 4, Ireland; 6 Institute for Global Food Security, Queens University Belfast, Belfast BT9 5DL, UK

**Keywords:** cell-penetrating peptides, milk, NF-κB, TNFα, macrophages

## Abstract

Milk-derived peptides are known to confer anti-inflammatory effects. We hypothesised that milk-derived cell-penetrating peptides might modulate inflammation in useful ways. Using computational techniques, we identified and synthesised peptides from the milk protein Alpha-S1-casein that were predicted to be cell-penetrating using a machine learning predictor. We modified the interpretation of the prediction results to consider the effects of histidine. Peptides were then selected for testing to determine their cell penetrability and anti-inflammatory effects using HeLa cells and J774.2 mouse macrophage cell lines. The selected peptides all showed cell penetrating behaviour, as judged using confocal microscopy of fluorescently labelled peptides. None of the peptides had an effect on either the NF-κB transcription factor or TNFα and IL-1β secretion. Thus, the identified milk-derived sequences have the ability to be internalised into the cell without affecting cell homeostatic mechanisms such as NF-κB activation. These peptides are worthy of further investigation for other potential bioactivities or as a naturally derived carrier to promote the cellular internalisation of other active peptides.

## 1. Introduction

Milk has evolved in close coevolution between the producing mother and the digesting newborn, and a number of bioactivities have been identified [1,2,3,4] for milk peptides, which may provide a means by which they can regulate various physiological functions in the offspring. The reported biological activities of milk-derived peptides include immunomodulatory, antimicrobial, analgesic, anticancer, antiviral, antioxidant, antithrombotic, opioid, anti-obesity, mineral-binding, anti-cytotoxic, antihypertensive and antiseptic properties [1,5,6,7,8,9,10,11]. Peptides are naturally released from milk proteins in the stomach and duodenum and may be released during food processing by hydrolysis using protease extracts of animal, plant or microbial origin, or using microbial fermentation [3,5,12,13,14,15,16]. Most of the identified milk bioactive peptides are cleavage products of the two primary milk protein fractions, namely, whey (β-lactoglobulin, α-lactalbumin, serum albumin, immunoglobulins and lactoferrin) and casein (α-, β- and κ-caseins) [2,17,18,19,20,21,22].

There is strong interest in developing milk extracts enriched for bioactive peptides, either for nutraceutical/pharmaceutical applications or to claim health-giving properties for food preparations [5,12,23,24,25]. Current target-specific screening approaches are designed to modulate extracellular receptors, such as angiotensin-1-converting enzymes and dipeptidyl peptidase 4 [26,27]. However, there are many potential intracellular targets, which can only be modulated by cell-penetrating peptides (CPPs), which manage to cross the cell membrane. Although CPPs present a promising potential in clinical use either as therapeutic agents in themselves or as a part of complexes between CPPs and cargoes, none have been approved for clinical use [28]. However, from a regulatory point of view, milk-derived preparations have the potential to possess more acceptable safety profiles.

One area of particular interest for milk is the modulation of inflammation. Anti-inflammatory effects of milk-derived peptides may occur via inhibition of MAP kinase signalling [29], down-regulation of lipopolysaccharide-induced cytokine production in monocytic cells [30,31], blocking excessive dendritic cell activation upon Toll-like receptor-induced inflammation, inhibition of the NF-κB pathway through a PPAR-γ dependent mechanism [32,33], downregulation of TNFα-induced inflammatory signalling [34], down-regulation of Janus kinase-signal transducer and activator of transcription (JAK-STAT) [35], and attenuation of IL-1β secretion [36]. Most of these pathways have both intracellular and extracellular components. While CPPs can be used as carriers to bring attached bioactive cargos into cells, there is also interest in CPPs which themselves have bioactivity, which have been referred to as bioportides [37]. CPPs can inhibit NF-kB and other targets crucial for the process of inflammation [38,39,40]; in the latter study, the positive charge of the pentameric peptide AIP6 was associated with both cell penetration and DNA binding activities (competing with NF-κB). It is possible that some of the inflammation-modulating effects of milk-derived peptides have evolved to facilitate the immune health of the neonate. Thus, milk peptides that act intracellularly or extracellularly represent a potential source of therapeutic components in the prevention of many chronic diseases that have an inflammatory component, such as cancer, rheumatoid arthritis, diabetes, cardiovascular disease, obesity, inflammatory bowel disease and asthma [4,41].

pH is important for CPP activity. Cell-penetrating peptides (CPPs) may penetrate cells via mechanisms such as oligomerisation/aggregation leading to pore formation, endocytosis and membrane lysis [38,42,43,44,45]. Positively charged arginine-rich CPPs bind to the negatively charged cell membrane, which allows them to enter cells [44]. Positively charged lysine-rich CPPs can form cationic liposomes that can fuse with the cell membrane and deliver their cargo into the cell [46]. Cell lytic peptides similarly often rely strongly on positive charges, and histidine can confer increased bioactivity on anti-cancer peptides in a pH-dependent manner [47] since histidine becomes more positively charged in low pH conditions. Most computational predictors of CPPs [48,49,50,51,52,53,54,55,56,57] are trained on known experimental datasets using machine learning approaches. However, these training sets are mainly derived from experiments that were performed at close to physiological intracellular pH. In contrast, the mammalian gut encounters a wide range of pH conditions, and there is no model specifically trained to allow for low pH conditions.

In the present study, we focused on one particular milk protein of interest, bovine α-S1-casein. We utilised a CPP predictor, CPPpred [58], to systematically survey all possible peptides in a given size range for their CPP potential. Given the importance of positively charged residues in cell-penetrating peptides, we were interested in manipulating the computational predictions in such a way that histidine is treated as more positively charged than would be normally considered by the trained predictor. Accordingly, we additionally modified the input sequences, replacing histidine with lysine. Peptides were predicted from a histidine-rich region of the protein and from another region. Representative peptides were then selected, synthesising the original milk peptides (rather than the lysine-substituted histidines, which were used only for the purposes of computational prediction) with and without fluorescent tags. We investigated the abilities of the peptides to enter cells and their ability to modulate inflammation.

## 2. Results

### 2.1. Prediction of Cell-Penetrating Peptide Sequences

We used CPPpred to predict the cell penetrating potential of 2087 potential peptides from alpha-S1-casein (Table S1). This assigns a score of 1 to very likely cell-penetrating peptides and 0 to those very unlikely to be cell-penetrating. As the majority of the peptides ranking highly under this selection criteria were from the same region of alpha-S1-casein (between residues 110 and 125; see Figures S1 and S2) (CPPpred scores > 0.60), we selected a number of peptides to get a suitable representation of the peptides nested within the span of this region (see Table 1). We noted that 282 of the 2087 peptides investigated contained histidine, which may be positively charged in certain pH contexts. Given that cell-penetrating peptides are often positively charged, we generated modified CPPpred scores for these peptides, substituting the histidines with lysines, purely for the purpose of prediction (Figure S3). We identified a region directly at the start of the protein which had the highest modified CPPpred scores among the 282 peptides. This region contains two histidines at the start of the mature protein. We selected two representative peptides from this region (Table 2). The native peptides were synthesised (i.e., with the histidines in the original sequence) with and without the 5-FAM fluorescent label using the linkages shown in Table 1 and Table 2. Two replicates of the experiments were performed as shown in Figure S4. While it would be of interest to test all the predicted peptides, for reasons of resource limitation, we selected a few representative peptides. These were chosen after visual inspection of the location and degree of overlap among different sets of peptides.

### 2.2. Internalisation of Peptides into Cells

**Figure 1:**
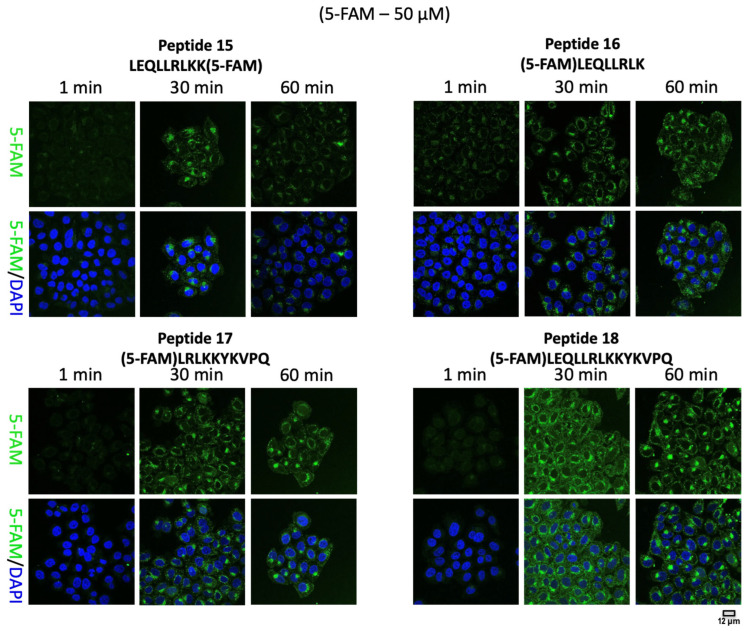
Representative confocal images of HeLa cells treated with 5-FAM (green) labelled synthetic peptides (peptides 15, 16, 17 and 18) at 1, 30 and 60 min. Nuclei are stained with 4′,6′-diamidino-2-phenylindole (DAPI) (blue).

**Figure 2:**
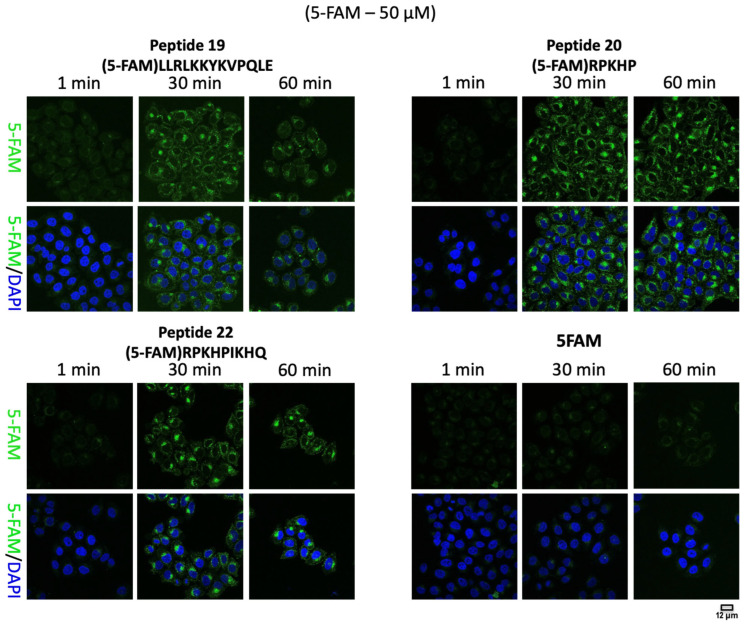
Representative confocal images of HeLa cells treated with 5-FAM (green) labelled-synthetic peptides (peptides 19, 20 and 22) and 5-FAM control at 1, 30 and 60 min. Nuclei are stained with 4′,6′-diamidino-2-phenylindole (DAPI) (blue).

When HeLa cells were treated with the 5-FAM-labelled synthetic peptides, the majority of cells demonstrated uptake of CPP into the cytoplasm, as determined by confocal microscopy (Figure 1 and Figure 2). Individual cells were identified by staining using 4′,6′-diamidino-2-phenylindole (DAPI), a dye that is commonly used to assess nuclear morphology. The fluorescent CPPs were clearly distinguished inside the cell at all time points examined. Also evident was that at earlier times after the addition of the peptide, the peptide could be seen at the extreme cell periphery, likely the plasma membrane, as well as having an intracellular punctate distribution. After longer incubation times, the pattern became concentrated towards the centre of the cell in a more juxta-nuclear pattern (Figure S4). This change in the intracellular distribution of the internalised peptides, from peripheral to juxta-nuclear, is what is classically observed during endocytosis, as molecules pass from early endosomes to late endosomes. Quantification of the confocal images suggested that although all the peptides tested were internalised into the cells, peptide 18 showed a relatively higher intracellular concentration (Figure S5).

To further establish the nature of the punctate pattern seen, these experiments were repeated. Peptides were allowed to accumulate for 60 min, but then the cells were fixed and immunostained for various markers of the endomembrane system. Distinct membrane structures containing peptides and the early endosome marker EEA1 as well as the late endosome/lysosome marker LAMP1 could be seen at this time point following internalisation (Figure S6). This observation would be consistent with the peptides being internalised through the endocytic pathway.

### 2.3. Effects on Inflammatory Markers and Toxicity

**Figure 3:**
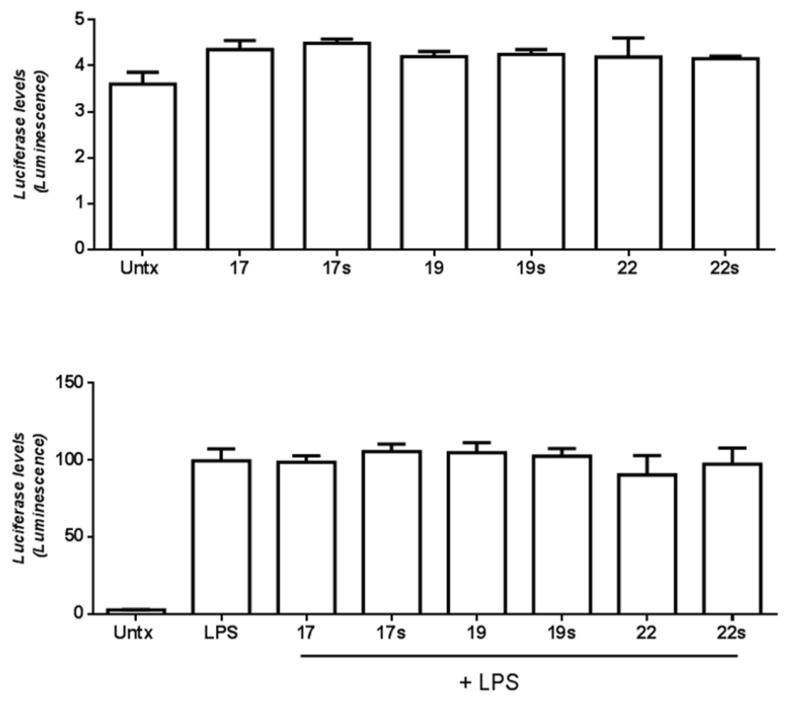
Top: Effect of peptides 17, 19 and 22 and scrambled peptides (suffix “s”) on NF-κB activity measured by luciferase assay without LPS stimulation; bottom left: NF-kB secretion by luciferase assay with LPS stimulation. Untx refers to untreated cells. Data represent mean ± SEM for *n* = 3 independent experiments.

**Figure 4:**
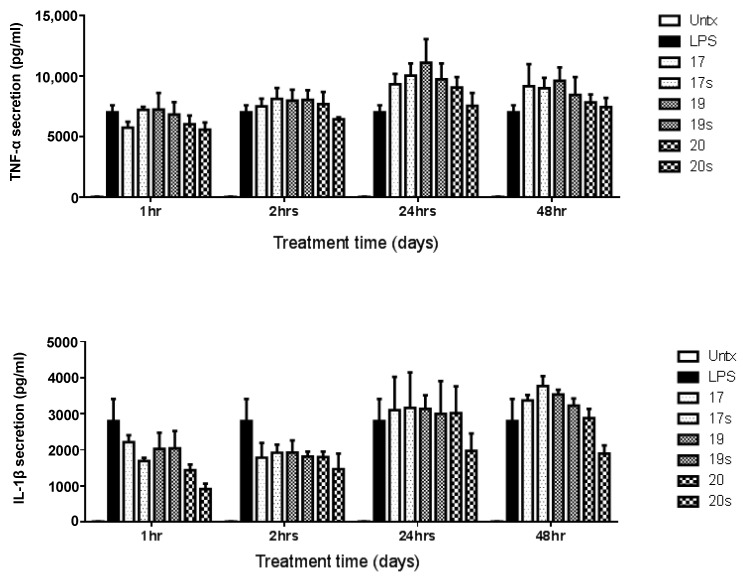
Top: Effects of peptides 17, 19, and 20 (and scrambled peptides denoted with suffix “s”) on TNFα secretion with LPS stimulation; bottom right: Effects of peptides on IL-1β secretion with LPS stimulation. Untx refers to untreated cells. Data represent mean ± SEM for *n* = 3 independent experiments.

We hypothesised that peptides may modulate an innate immune response, in cells such as macrophages. However, we observed no effect of the tested peptides 17, 19 or 22 on NF-κB activity, measured by a luciferase reporter assay as either in the presence or absence of LPS stimulation (Figure 3). Similarly, there was little effect of the peptides tested (17, 19 and 20) on TNFα or IL-1β secretion from macrophages over an extensive time course, at 1-, 2-, 24- or 48-h time points (Figure 4). Peptide 20 showed some marginal decrease in IL-1β secretion at the early time-point, but this was not significant. Very similar results were found for scrambled peptides (Figure 3). The peptides displayed no toxicity on cells when assessed using an MTT assay, a standard approach for measuring cell metabolic activity (Figure S6).

## 3. Discussion

This study identified a number of alpha-S1-casein-derived peptides that are cell-penetrating and that do not have pro-inflammatory or cell toxicity effects. This raises the interesting possibility that protein fragments released on digestion have the potential to enter cells and induce signalling effects without triggering unwanted immune responses during milk digestion. While the peptides may typically be readily digested by trypsin in the gut lumen, it is possible that they may enter cells. Thus, these milk-derived peptides may have a useful safety or functional profile in contrast to other cell-penetrating peptides which are often used to carry a cargo into a target cell.

It is of interest to consider what functions these cell-penetrating peptides could confer. CPPs are typically 5–30 amino acids in length, positively charged and partly hydrophobic [59], and such properties are shared by many antimicrobial peptides. NF-κB activation is one of the most important inflammatory pathways governing cytokine secretion in innate immunity. Cell-penetrating peptides have previously been shown to have the ability to inhibit NF-κB activity. One of the most commonly used CPPs, Tat, has demonstrated immune-modulating abilities. Tat demonstrated the ability to reduce cytokine secretion induced by phorbol 12, 13-dibutyrate (PDBu) including TNFα and IL-6 in vitro [60]. Degradation of the NF-κB transcription factor inhibitor, IκBα, was also reduced with Tat pre-treatment. Another cell-penetrating peptide, pVEC, significantly inhibited LPS-induced nitric oxide production and TNFα secretion from mouse macrophages [61]. Small cationic peptides with anti-microbial activity have also demonstrated the ability to reduce LPS-induced TNFα secretion [62].

Interestingly, while demonstrating significant cell-penetrating ability, none of the peptides had a significant effect on TNFα or IL-1β secretion from J774.2 mouse macrophages. Both the regions of alpha-S1-casein from which the cell-penetrating peptides identified here are overlapped by known antimicrobial peptides. The sequence LRLKKYKVPQL (contained within peptide 19) demonstrated antibacterial activity in inoculated fresh pear juice and against Gram-positive and Gram-negative bacteria [63,64]. LEQLLRLKKY, HIQKEDVPSERYLGYLEQLLRLKKYK and HIQKEDVPSERYL-GYLEQLLRLKK, all containing the cell-penetrating sequence LEQLLRLKK, have also been identified in a virtual screening of a milk peptide database for the identification of food-derived anti-microbial peptides [65]. A number of peptides overlap with the two tested histidine-containing cell-penetrating peptides (peptides 20 and 22, RPKHP and RPKHPIKHQ). The anti-microbial peptide isracidin (RPKHPIKHQGLPQEVLNENLLRF) from the first 23 residues of α-s1-casein has significant anti-bacterial activity versus *E. coli* and *E. sakazakii* [66,67]. RPKHPIK was present in water-soluble extracts of several Italian cheese varieties that demonstrated anti-bacterial activity towards both Gram-positive and Gram-negative bacteria [68]. Peptide 22 (RPKHPIKHQ) is present in cheddar cheese [69,70,71] and has demonstrated ACE-inhibitory activity in vitro [71], while RPKHPI, released by culturing casein with *Lactobacillus helveticus*, has anti-microbial and ACE-inhibitory activity [72]. Given the strong biophysical overlap between cell-penetrating and antimicrobial peptides, the antimicrobial effects of cell-penetrating peptides may not be their primary target, so it is worthwhile considering alternative potential primary intracellular targets.

One interesting feature of the two cell-penetrating peptides that contain histidine is that they should be less prone to trypsin digestion in the intestine than an equivalent cell-penetrating peptide replacing the histidines with lysine or arginine, and the presence of proline at the second position of these two peptides may contribute to their resistance to digestion by the ACE enzyme, which is highly expressed in brush border membranes. Thus, they may be well-adapted to the proteolytic environment of the gut.

While there are a large number of histidine-containing peptide sequences on the database of cell-penetrating peptides CPPSite 2.0 [73], most of these contain the other positively charged residues lysine and arginine which may be conferring the cell penetrating potential at the physiological pH [74]. Our strategy of replacing histidine with lysine in the predictor assumed that histidine’s positive charge under certain pH conditions could play a role in penetration. When we tested the selected histidine-rich peptides, we noted that under standard pH conditions, the two histidine-containing peptides exhibited cell-penetrating properties. This experimental validation provides some support for our approach of designing positively charged cell-penetrating peptides that may be resistant to breakdown by endoproteases that cleave lysine and arginine, such as trypsin. More generally, this strategy points to potential peptide design opportunities that extrapolate beyond known training sets through the replacement of residues in the initial predicted sequences with those of amino acids with overlapping features, either making specific targeted replacements as we did here or more generally creating larger datasets of potential binders for subsequent experimental or computation validation or refinement, such as might be achieved by the use of peptide generative models.

The choice of HeLa cells for peptide penetration in this report was based on the ease of visualising peptides in these cells. Indeed, these experiments allowed us to observe that immediately following internalisation, the peptides followed a classic redistribution from the cell periphery (where early endosomes are in high abundance) to a more juxta-nuclear location (where more acidic late endosomes are located). We also assessed the effect of peptide internalisation on three different inflammatory responses, this time in more physiologically relevant cells. Indeed, penetration into immune cells, which may be less negatively charged, is clinically relevant. However, we cannot rule out that these peptides may impact inflammatory processes outside of the three pathways investigated. It would be intriguing to investigate the cell-penetrating ability of these peptides within a more physiologically relevant context. An example of this would be tissue imaging to visualise the uptake of peptides in different cell types of the gut in fresh biopsy samples, investigated under different pH conditions, reflecting the fluctuating pH states in the stomach and duodenum. Other aspects that influence the ability of the peptides to enter cell types could also be experimentally manipulated (physical barriers, protease activities close to the gut wall, closed state of gut tight junctions). Of particular interest would be the ability of milk-derived peptides to enter the enteroendocrine cells such as the L cells of the intestine, which play an important regulatory role in determining various aspects of gut function. Milk peptides are capable of regulating key endocrine functions of these cells [75].

The approach of systematically investigating all possible overlapping peptides of varying lengths contained within a protein sequence is another feature of our work that is likely to have more general applicability. It is more general than most experimental approaches, which typically use a fixed peptide window size, whether those study designs are based on peptide arrays of biologically selected peptides [76], on proteome-wide phage display of peptides whose sequences are derived from windows within proteins [77], or on combinatorial phage display [78]. The variable window strategy we employed is difficult to formally validate, as there is not a large body of experimental evidence exactly matching the computational peptide size distributions. It will be interesting to see if other applications of this method provide evidence of some practical utility such as this work here indicates.

## 4. Materials and Methods

### 4.1. Computational Derivation of Peptides

The amino acid sequence of Bos taurus Alpha-S1-casein was retrieved from the UniProt database [79] with the accession code P02662. Python scripts were used to generate all possible peptides of length between 5 and 30 amino acids from the mature region of the protein, excluding the signal peptide (Figure S1). The potential cell-penetrability of the generated peptides was determined using the CPPred software (http://distilldeep.ucd.ie/CPPpred/) [58] and bioactivity predicted using Peptide Ranker [80,81]. CPPpred is a computational web server that predicts CPPs using an N-to-1 neural network. Users can input one or more peptide sequences, between 5 and 30 amino acids in length, and the server returns a prediction of how likely each peptide is to have the potential to be cell-penetrating. All generated peptides were uploaded to the CPPpred web server, and the corresponding CPPpred scores ranging between 0 and 1 (higher scores suggested higher probability of cell penetrability) were recorded and presented in the Supplementary Files. Similarly, Peptide Ranker employs a novel N-to-1 neural network to predict bioactive peptides based on input peptide sequences. Generated peptides were submitted to Peptide Ranker, which returned results for each peptide by ranking the probability that the given peptide would be bioactive. The generated Peptide Ranker score for each peptide was also recorded and presented in the Supplementary Files.

### 4.2. Peptide Synthesis

Peptides were synthesised with and without an amine-reactive fluorescent label (5-FAM, 5-Carboxyfluorescein) to determine the ability of each peptide to traverse through cellular membranes (peptide sequences (LRLKKYKVPQ, RVPLKKQYLK, LLRLKKYKVPQLE, LEVKYRLKLPKQL, RPKHP, PHKPR). 5-FAM was attached at the N-terminus for all but one of the CPPs. In the case of peptide 15, the 5-FAM label was attached to an additional lysine at the C-terminus, to investigate any effects on cell permeability that the orientation of labelling might have, for comparison with the same peptide (peptide 16) with an N-terminal label. Peptides were synthesised by the peptide supplier Peptide2.0 with high purity (>99%), as determined by HPLC analysis (220 nm, C18, linear gradient).

### 4.3. Cell Culture

Wild-type HeLa cells (ATCC CCL2) were grown in Dulbecco’s modified Eagle’s medium (Life Technologies, Carlsbad, CA, USA) supplemented with 10% heat-inactivated foetal bovine serum (FBS) (PAA Laboratories, Cölbe, Germany) and 1% glutamine (Life Technologies, Carlsbad, CA, USA). The NIH3T3 cell line stably transfected with an NF-κB luciferase reporter vector was purchased from Panomics (Affymetric Inc., Santa Clara, CA, USA), and cells were plated at 2.5 × 105 cells/mL in DMEM containing 10% FBS, 1% Pen-Strep and 100 μg/mL hygromycin.

The J774.2 mouse macrophage cell line was obtained from the European Collection of Cell Cultures (ECACC). Cultures were maintained between 3–9 × 105 cells/mL at 70–90% confluency in T-75 culture flasks in complete media. Cells were passaged 2–3 days before use and detached by tapping sharply on the flask and pipetting up and down several times. Cells were centrifuged at 1200 rpm for 5 min; the pellet was resuspended in 12 mL DMEM and cells counted using a haemocytometer.

### 4.4. Confocal Image Acquisition

Confocal images (1024 × 1024 pixels) were acquired with an Olympus FV1000 confocal microscope equipped with a 60×, 1.35 NA oil immersion objective and imaged with laser lines appropriate for the various fluorophores. Cells for confocal imaging were prepared using fixation in 3% PFA followed by incubation with 0.2 μg/mL DAPI at room temperature for staining cell nuclei.

### 4.5. NF-kB Expression in Fibroblasts

Cells were treated with 50 μM peptides or with scrambled versions of the same peptides for 24 h before stimulation with/without 10 ng/mL LPS for 6 h. Cells were washed with PBS, and 50 µL 1X Reporter Lysis Buffer (Promega Corporation, Madison, WI, USA) buffer was added to the lyse cells. An amount of 10 μL of cell lysate was transferred to a 96-well fluorescence sterile polystyrene Fluoro Nunc white plate (Fisher Scientific, Loughborough, UK). A total of 50 μL of Luciferase Assay Reagent was added to the cell lysate, and luminescence was measured at 560 nm using a luminometer. The Luciferase Assay monitors and quantifies the binding activity of the transcription factor NF-kB.

### 4.6. TNF and IL-1β Secretion from Macrophages

J774.2 macrophages were plated at 1.2 × 105 cells/mL in DMEM containing 10% FBS and 1% Pen-Strep. Cells were treated with 50 μM of peptide and scrambled control peptides for the time indicated before stimulation with 10 ng/mL LPS for 4 h. Cytokine concentrations in cell supernatants were measured using ELISA Duoset kits (R & D systems, Abingdon, UK) for mouse TNFα and IL-1β, according to the manufacturer’s instructions. TNF and IL-1β are cytokines that are secreted by macrophages in response to various stimuli, hence their use in this study to assess the anti-inflammatory potential of the identified peptides.

### 4.7. MTT Assay

MTT assay was performed in accordance with previously published reports [82]. The assay involves the conversion of the water-soluble MTT(3-(4,5-dimethylthiazol-2-yl)-2,5-diphenyltetrazolium bromide) to an insoluble formazan. The formazan is then solubilised, and the concentration is determined with optical density absorbance at 570 nm. The optical density of the formazan solution is proportional to the number of living cells in the culture, as only living cells have the ability to metabolise MTT into formazan. The MTT assay can be used as an indicator of toxicity because changes in cell viability and proliferation can be a sign of cellular stress or damage caused by toxic molecules. Cells exposed to toxic molecules may have their metabolic activity and ability to reduce MTT affected, resulting in a decrease in the amount of formazan produced. Thus, a decrease in MTT assay results may indicate that a compound is toxic to the cells (Figure S7).

## 5. Conclusions

We combined computational and experimental methods to identify and synthesise peptides from the milk protein alpha-S1-casein. Using computational techniques, we identified and synthesised bioactive peptides from the milk protein, alpha-S1-casein. We subsequently investigated the cell-penetrating ability and anti-inflammatory effects of the peptides using confocal microscopy analysis and determined the effects of the peptides on known inflammatory markers via in vitro assays using HeLa cells and J774.2 mouse macrophage cell lines. Results showed the cell-penetrability of the peptides evidenced by an observed fluorescence in the cytoplasm of cells treated with peptides that were synthesised with an amine-reactive fluorescent label. The identified peptides, however, demonstrated no effect on the activity of the NF-κB transcription factor as well as the secretion of TNFα and IL-1β, which are all known inflammatory markers. The peptides also showed no effect on markers of toxicity. It could thus be concluded that the milk-derived sequences have the ability to be internalised into the cell without affecting cell homeostatic mechanisms such as NF-κB activation and could be further investigated towards the design of novel therapeutic agents.

## Figures and Tables

**Table 1:** Selected peptides of interest from alpha-S1-casein (110–125).

Peptide No.	Peptide Sequence	CPPpred Score
15	LEQLLRLKK-(5-FAM)	0.792
16	(5-FAM)-LEQLLRLK	0.751
17	(5-FAM)-LRLKKYKVPQ	0.727
18	(5-FAM)-LEQLLRLKKYKVPQ	0.678
19	(5-FAM)-LLRLKKYKVPQLE	0.666

**Table 2:** Selected histidine-containing peptides of interest from those identified with high modified CPPpred score (modified by substituting histidines with lysines).

Peptide No.	Modified CPPpred Score	Peptide Sequence	Original CPPpred Score
20	0.772	(5-FAM)-RPKHP	0.514
22	0.674	(5-FAM)-RPKHPIKHQ	0.324

## Data Availability

Not applicable.

## Supplementary Materials

The following supporting information can be downloaded at: https://www.mdpi.com/article/10.3390/molecules28196999/s1, Figure S1: Peptigram-generated peptide alignment map of identified peptides from the full alpha-S1-casein protein (P02662) of Bos taurus (residues 1-214). The alignment map shows the sequence of alpha-S1-casein with each of the identified peptides mapped to their associated position. Top: sequence alignment with Homo Sapiens alpha-S1-casein retrieved by Proviz. Peptides with CPPpred scores greater than 0.5 (from Table 1) are displayed in green in the next pane. The majority of the peptides ranking highly under this selection criteria are shown to occur in the region of residues 110–125 of alpha-S1-casein. The trypsin cleavage site panel displays sites for trypsin cleavage in brown. The mobiDB panel highlights regions annotated to be part of a structurally disordered region. Figure S2: Bar chart showing the CPPred scores of Alpha-S1 casein-derived peptides with score >0.60. Highlighted in red are peptides predicted and synthesised in this report. Figure S3: Bar chart showing the CPPred scores of H to K substituted Alpha-S1 casein-derived peptides with scores >0.50. Highlighted in red are peptides predicted and synthesised in this report (RPKKP, RPKKPIKKQ). Figure S4: Quantification of normalized fluorescence intensity of intracellularly located 5-FAM labeled peptides at 1 min, 30 min, and 60 min incubation. Figure S5: Upper panels show intracellular localization of (5-FAM)-peptide 18 (green) at various times after addition to HeLa cells. Cells were also stained with DAPI (blue). Lower panels show zoomed regions from the 30 min and 60 min incubations, allowing visualization of the peptides at the plasma membrane, in punctate structures, and also in a juxta-nuclear pattern. Figure S6: Representative confocal images of HeLa cells treated for 60 min with 5-FAM (green) labelled synthetic peptide 18. Cells were immunostained for either the early endosome marker EEA1 (red, upper panels) or the late endosome/lysosome marker LAMP1 (red, lower panels). Nuclei are stained with Hoechst 33342 (blue). Arrows indicate green and red co-localising structures, which appear as yellow. Figure S7: MTT cytotoxicity assay absorbance. Table S1: Alpha-S1 casein-derived peptides and H to K substituted alpha-S1 casein-derived peptides.

## Abbreviations

CPP	cell-penetrating peptides
siRNA	small interfering RNA
NF-kB	nuclear factor kappa B
JAK-STAT	Janus kinase-signal transducer and activator of transcription
DAPI	4′,6′-diamidino-2-phenylindole
EEA1	Early Endosomal Antigen 1
5-FAM	5-carboxyfluorescein
TNFα	tumour necrosis factor alpha
IL-1β	interleukin-1 beta
LPS	lipopolysaccharide
PDBu	phorbol 12, 13-dibutyrate
ACE	Angiotensin-converting enzyme
FBS	foetal bovine serum
ECACC	European Collection of Cell Cultures
MTT	3-(4,5-dimethylthiazol-2-yl)-2,5-diphenyltetrazolium bromide
