# Supplementary material for: Cell-Penetrating Milk-Derived Peptides with a Non-Inflammatory Profile
Source: Molecules. 2023 Oct 9;28(19):6999. doi: 10.3390/molecules28196999 (PMC10574647; doi:10.3390/molecules28196999)
Supplement: Supplementary file 1 [file molecules-28-06999-s001.zip › molecules-2242148-supplementary.pdf]

Cell-penetrating milk-derived peptide with a non-inflammatory profile.

Clement Agoni[1,2,6#], Ilias Stavropoulos[1,2#], Anna Kirwan[1,5#], Margaritha M. Mysior[1,5], Therese Holton[1,3], Tilen Kranjc[1,5], Jeremy C. Simpson[1,5], Helen M. Roche[1,4], Denis C. Shields[1,2]

Supplementary Figures

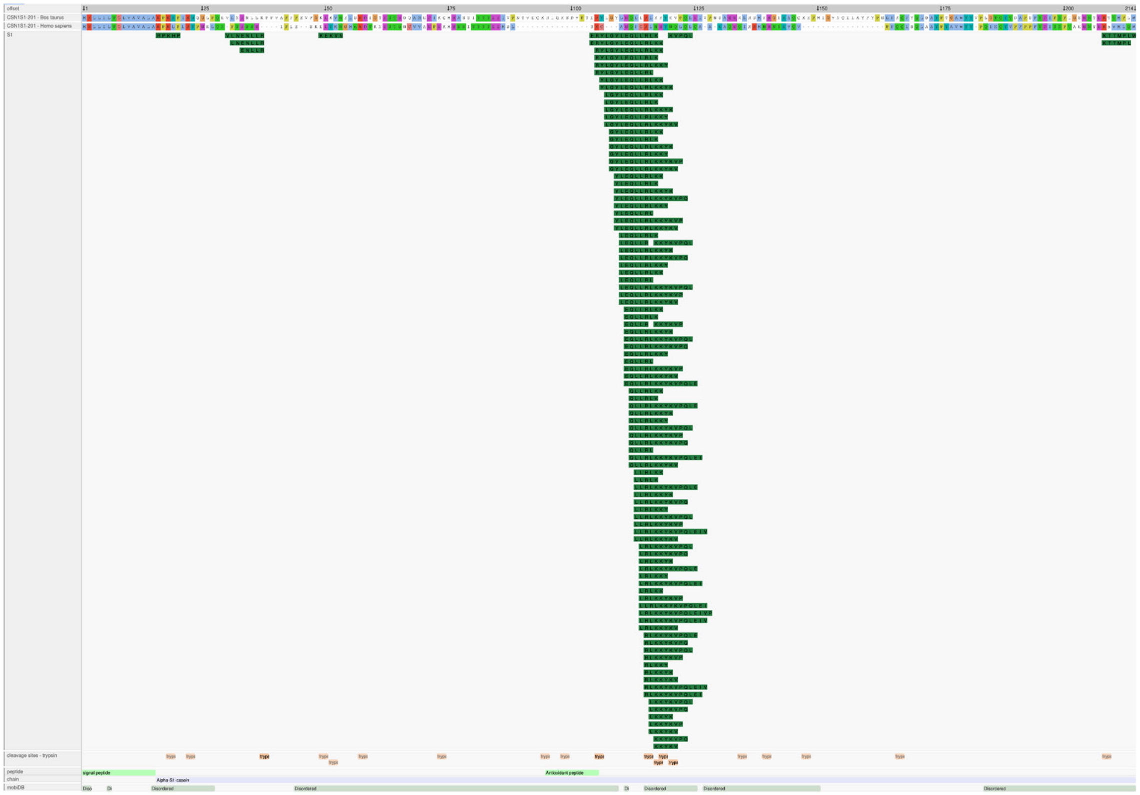

Figure S1: Peptigram-generated peptide alignment map of identified peptides, from the full alpha-S1-casein protein (P02662) of *Bos taurus* (residues 1-214). The alignment map shows the sequence of alpha-S1-casein with each of the identified peptides mapped to their associated position. Top: sequence alignment with *Homo Sapiens* alpha-S1-casein retrieved by Proviz. Peptides with CPPpred scores greater than 0.5 (from Table 1) are displayed in green in the next pane. The majority of the peptides ranking highly under this selection criteria are shown to occur between residues 110-125 region of alpha-S1-casein. The trypsin cleavage site panel displays sites for trypsin cleavage in brown. The mobiDB panel highlights regions annotated to be part of a structurally disordered region.

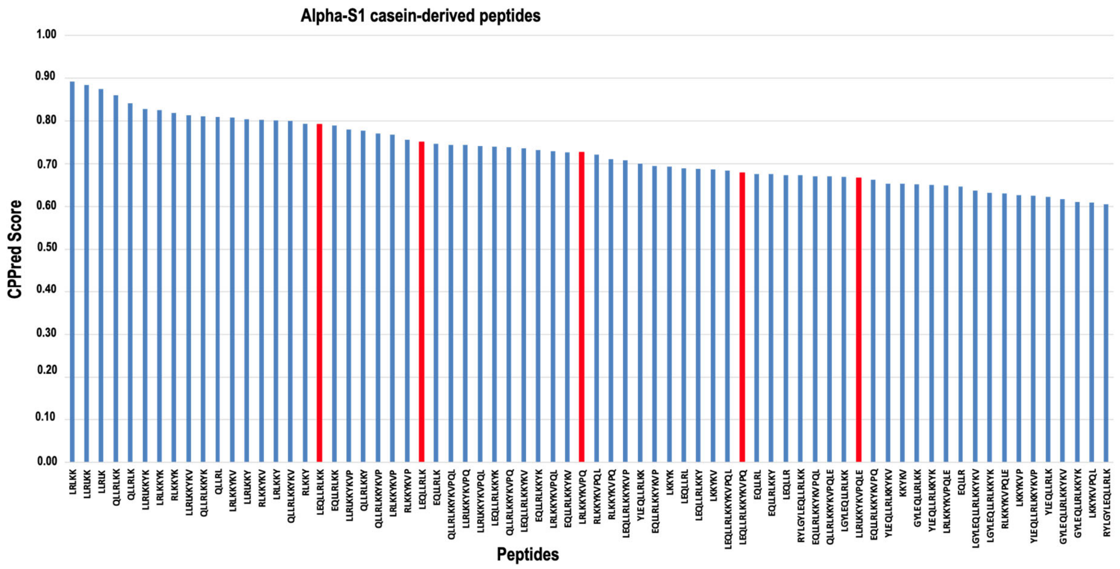

Figure S2: Bar chart showing the CPPred scores of Alpha-S1 casein-derived peptides with score >0.60. Highlighted in red are peptides that were predicted and synthesized in this report.

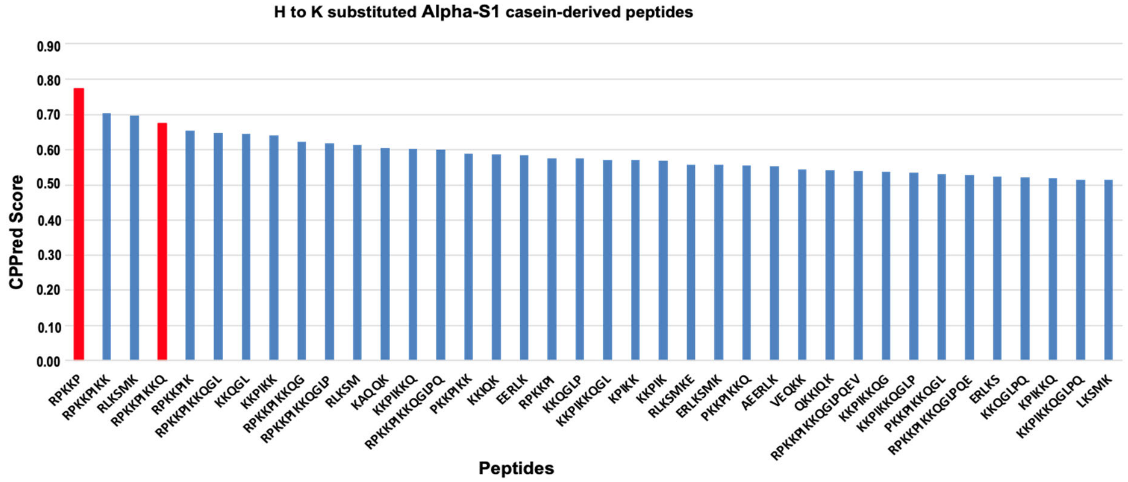

Figure S3: Bar chart showing the CPPred scores of H to K substituted Alpha-S1 casein-derived peptides with scores >0.50. Highlighted in red are peptides predicted and synthesized in this report (RPKKP, RPKKPIKKQ).

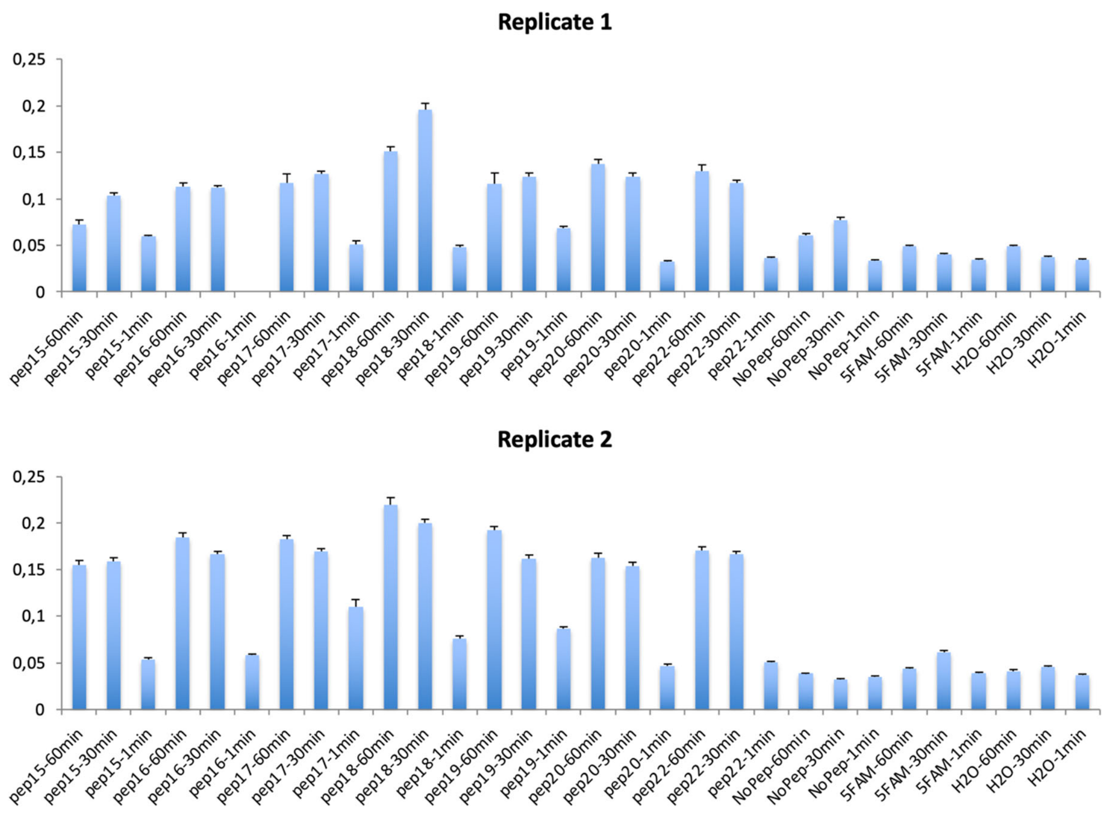

Figure S4: Quantification of normalized fluorescence intensity of intracellularly located 5-FAM labeled peptides at 1 minute, 30 minutes, and 60 minutes incubation.

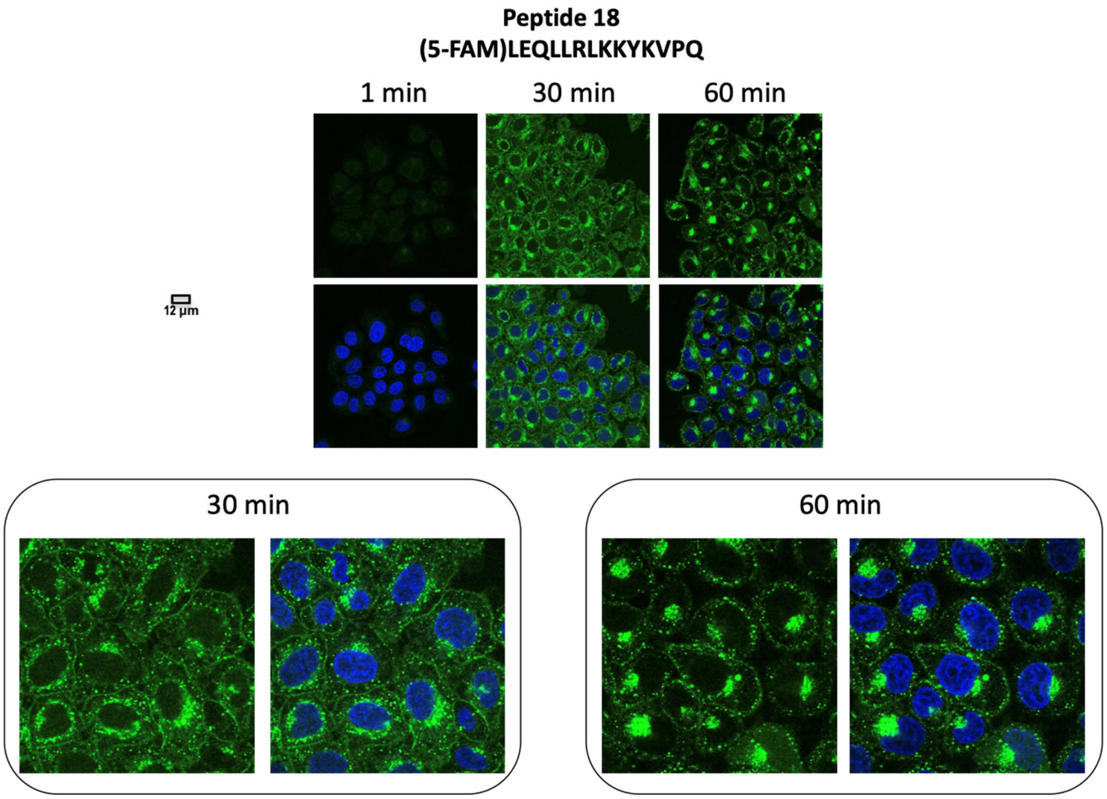

Figure S5: Upper panels show intracellular localization of (5-FAM)-peptide 18 (green) at various times after addition to HeLa cells. Cells were also stained with DAPI (blue). Lower panels show zoomed regions from the 30 min and 60 min incubations, allowing visualization of the peptides at the plasma membrane, in punctate structures, and also in a juxta-nuclear pattern.

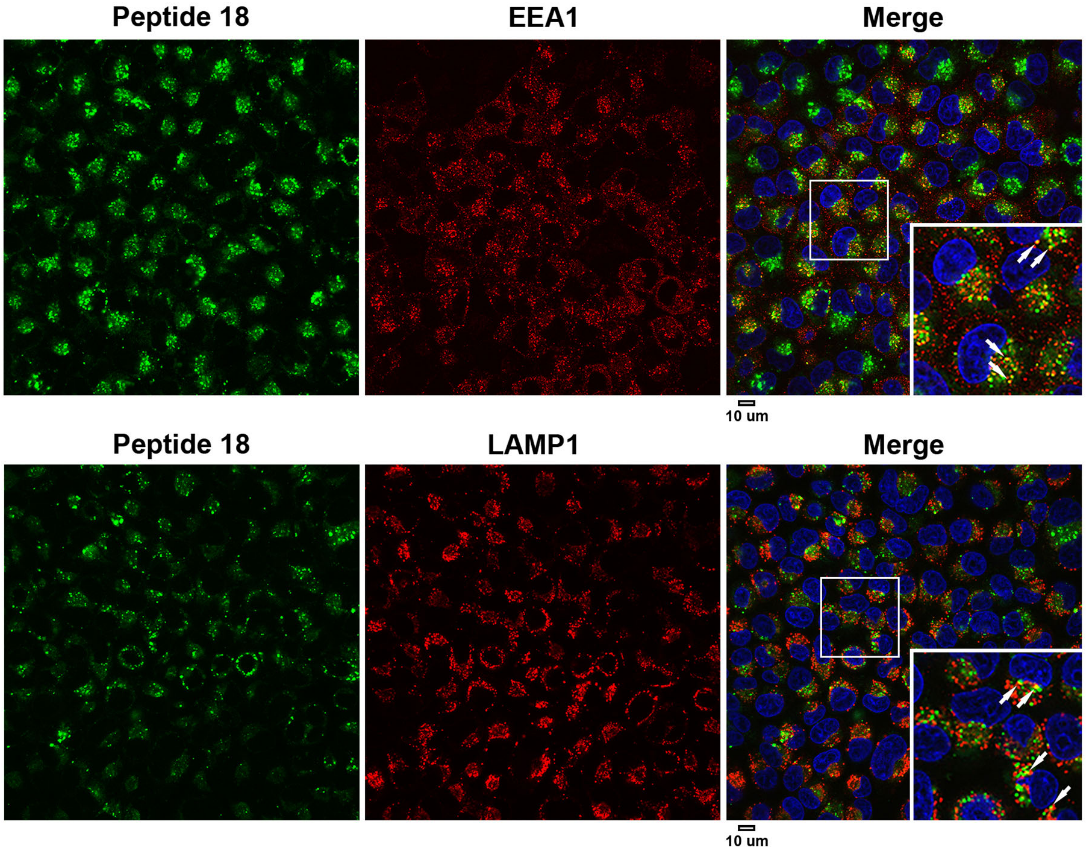

Figure S6: Representative confocal images of HeLa cells treated for 60 minutes with 5-FAM (green) labelled synthetic peptide 18. Cells were immunostained for either the early endosome marker EEA1 (red, upper panels) or the late endosome / lysosome marker LAMP1 (red, lower panels). Nuclei are stained with Hoechst 33342 (blue). Arrows indicate green and red co-localizing structures, which appear as yellow.

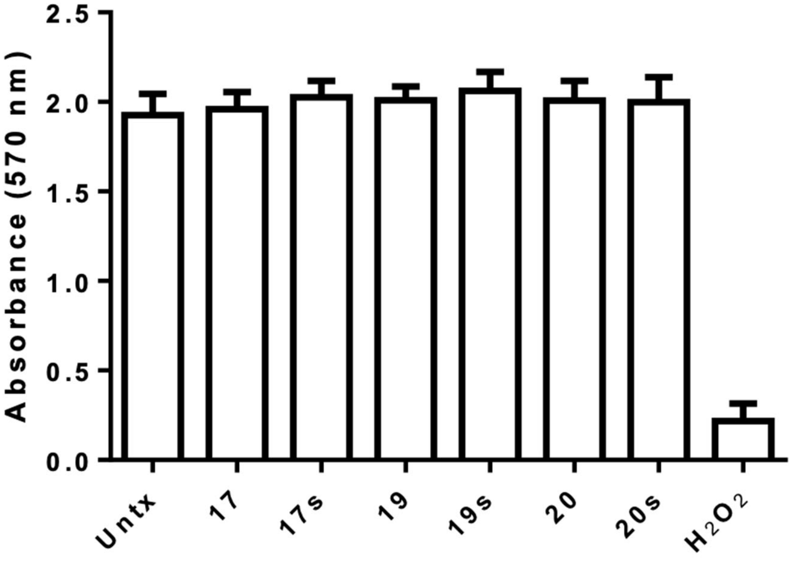

Figure S7: MTT cytotoxicity assay absorbance.
